# High N-Terminal Pro-B-Type Natriuretic Peptide Levels Are Associated with Reduced Heart Rate Variability in Acute Myocardial Infarction

**DOI:** 10.1371/journal.pone.0044677

**Published:** 2012-10-12

**Authors:** Luc Lorgis, Daniel Moreau, Laurent Mock, Bernadette Daumas, Daniel Potard, Claude Touzery, Yves Cottin, Marianne Zeller

**Affiliations:** 1 Centre de Cardiologie, CHU Dijon, Dijon, France; 2 Service d'Exploration Fonctionnelle, CHU Dijon, Dijon, France; 3 Laboratoire de Physiopathologie et Pharmacologie Cardiométaboliques, INSERM UMR 866, UFR Médecine, Université de Bourgogne, Dijon, France; 4 Service de Cardiologie, Clinique de Fontaine les Dijon, Fontaine les Dijon, France; Albert Einstein College of Medicine, United States of America

## Abstract

**Aim:**

We investigated the relationships between the autonomic nervous system, as assessed by heart rate variability (HRV) and levels of N-terminal Pro-B-type Natriuretic Peptide (Nt-proBNP) in patients with acute myocardial infarction (MI).

**Methods and Results:**

The mean of standard deviation of RR intervals (SDNN), the percentage of RR intervals with >50 ms variation (pNN50), square root of mean squared differences of successive RR intervals (rMSSD), and frequency domain parameters (total power (TP), high frequency and low frequency power ratio (LF/HF)) were assessed by 24 h Holter ECG monitoring. 1018 consecutive patients admitted <24 h for an acute MI were included. Plasma Nt-proBNP (Elecsys, Roche) was measured from blood samples taken on admission. The median (IQR) Nt-proBNP level was 681(159–2432) pmol/L. Patients with the highest quartile of Nt-proBNP were older, with higher rate of risk factors and lower ejection fraction. The highest Nt-proBNP quartile group had the lowest SDNN, LF/HF and total power but similar pNN50 and rMSSD levels. Nt-proBNP levels correlated negatively with SDNN (r = −0.19, p<0.001), LF/HF (r = −0.37, p<0.001), and LF (r = −0.29, p<0.001) but not HF (r = −0.043, p = 0.172). Multiple regression analysis showed that plasma propeptide levels remained predictive of LF/HF (B(SE) = −0.065(0.015), p<0.001)), even after adjustment for confounders.

**Conclusions:**

In conclusion, our population-based study highlights the importance of Nt-proBNP levels to predict decreased HRV after acute MI.

## Introduction

After acute myocardial infarction (MI), B-type natriuretic peptide (BNP) and the N-Terminal fraction of its propeptide (Nt-proBNP) are major prognostic factors, independently of left ventricular ejection fraction (LVEF). [1], [2] Modulation of Nt-proBNP is multifactorial, depending on left ventricular dysfunction, remodeling, on left intraventricular pressure, and residual myocardial ischemia. [3], [4] Left ventricular remodeling is a complex process affected by many factors notably the autonomous nervous system (ANS) through sympathetic activation. [5]

ECG Holter analysis is a validated non-invasive approach to evaluate the level of sympathetic and vagal tone. Loss of ANS balance frequently associated with coronary artery disease, is characterized by a fall in vagal modulation and a rise in sympathetic modulation. Heart Rate Variability (HRV) reflects cardiovascular response to the ANS. After MI, reduced HRV is an independent predictive factor of mortality and sudden cardiac death. [6], [7] Experimental studies in animals and humans strikingly showed that BNP infusion affects activity of the ANS through a decrease in sympathetic activity. [8], [9]

However, the relationship between plasma levels of Nt-Pro-BNP and ANS evaluated by Holter ECG analysis has never been fully explored, in particular in acute MI.

In a large prospective study, we set out to analyze the relationship between ANS, evaluated by the high and low frequency spectral components of HRV and the level of Nt-ProBNP at the acute phase of MI.

## Methods

### Patients

The participants were recruited from the RICO (observatoiRe des Infarctus de Côte-d'Or) database, a French regional survey for acute MI. [10] Briefly, RICO survey collects data from all the consecutive patients hospitalized <24 h for acute MI in all public centres or privately funded hospitals of one eastern region. The present study included all the consecutive patients admitted between 1^st^ July 2001 and 31^st^ March 2008 who underwent a 24-hour Holter ECG recording during the hospitalization. Patients with a pacemaker (n = 8), atrial fibrillation (n = 83) or heart rate dysfunction (n = 6) were excluded. The present study complied with the Declaration of Helsinki and was approved by the ethics committee of University Hospital of Dijon. Each patient gave written consent before participation.

### Data collection

Cardiovascular risk factors, hemodynamic parameters, i.e. heart rate (HR), systolic (SBP) and diastolic (DBP) blood pressure, Killip class, history of MI and acute treatments were prospectively collected. LVEF was determined by echocardiography using the Simpson method at 3±1 days.

### Biological data

Nt-proBNP levels were measured from blood samples taken at admission, kept at room temperature for 30 min then centrifuged at 2500 rpm at 4°C for 10 min. The median time from symptom onset to blood sampling was 180 (85–466) min. Plasma level of Nt-proBNP was determined by chemoluminescence (Elecsys 2010, Roche). Inter and intra-assay coefficient of variation were both <3%. The lowest detection limit of the assay was at 0.6 pmol/L. The limit for the detection of cross-reactivity with the other natriuretic peptides (ANP, BNP and CNP) was less than 0.01%. Peak Creatine Kinase (CK) was determined by blood sampling at 8-hour intervals during the first 48 h (Dimension analyzer, Roche). Creatinine clearance was calculated from plasma creatinine levels assessed from blood sample taken on admission. [11]

### Holter ECG data

24-hour (Holter) ECG was performed at 5±2 days. Long ECG tracing were recorded and analysed by two experienced observers using a Syneflash digital recorder Holter (Ela medical and Spieder Viers, le Plessis Robinson, France), with seven surface electrodes signals (acquisition sampling rate : 1000 Hz). After classifying the QRS morphology, the RR intervals (longest and shortest) were confirmed manually until no QRS sequences were incorrectly labeled. Only sequences with normal QRS characteristics were analyzed for HRV study.

Analysis of the temporal domain analyzed the indexes 1) rMSSD: root mean square successive difference between each value and the preceding value. 2) pNN50: % of successive intervals in which the difference exceeds 50 ms. 3) SDNN: standard deviation of all of the RR intervals between two normal QRS complexes.

Spectral analysis used the Fast Fourier Transform to convert the different successive RR intervals in the frequency domain. Low frequencies (LF, between 0.04 and 0.15 Hz) are affected by both vagal and sympathetic activity, whereas high frequencies (HF, between 0.15 and 0.4 Hz) are affected by vagal tone. The LF/HF ratio is therefore considered an indicator of sympathovagal balance; Oscillations in very low frequencies VLF (range 0.00 to 0.04 Hz) reflect peripheral vasomotor regulation. Total power (TP), combining the sum of all of the frequencies, is a global measure ANS activity.

### Statistical analysis

Data are expressed as medians (1^st^–3^rd^ quartile) or percentages (n (%)). Categorical variables were compared using the Chi-2 test and continuous variables using ANOVA. A correlation analysis was carried out using a Spearman or Pearson test. We used backward multivariate linear regression analysis to predict LF/HF. Variables associated with the LF/HF ratio in univariate analysis were included as covariables in the model (inclusion at 1%, and an exclusion at 5%). For not normal distribution, variables were log-transformed. The best linear adjustment was selected for inclusion in the model. Analyses were performed using SPSS 12.0 (SPSS Inc, Illinois, USA).

## Results

The baseline characteristics classified according to Nt-proBNP quartiles are presented in table 1. Among the 1018 participants, the median Nt-proBNP level was at 682 (159–2432) pg/ml. Patients with the highest Nt-proBNP quartile were almost 20 y older and with a higher rate of CV risk factors, except for smoking, and history than patients from the lowest quartiles. Patients from the highest quartile were also characterized by increased heart rate, lower SBP levels on admission and higher rate of anterior infarction. With increasing levels of Nt-proBNP, LVEF gradually decreased, while patients with HF increased. Beta-blockers were less given in patients with the highest levels of the propeptide, while amiodarone was more used. Infarct size, as assessed by biological markers (i.e. CK), was lower in the highest quartiles of propeptide.

**Table 1:** Characteristics of the study population according to Nt-proBNP quartiles.

	Q1	Q2	Q3	Q4	
	N = 254	N = 255	N = 255	N = 254	
Median Nt-proBNP (25^th^–75^th^), pg/ml	70 (39–107)	356 (238–479)	1238 (945–1640)	5605 (3451–11284)	p
Age (years)	55 (47–64)	64(52.5–73)	65(54–75)	76(65.25–81)	<0.001
Sex female	37(15%)	53(21%)	77(30%)	120(47%)	<0.001
Hypertension	78(31%)	129(51%)	132(52%)	161(63%)	<0.001
Diabetes	44(17%)	41(16%)	56(22%)	75(30%)	0.001
Body mass index (kg/m^2^)	26(24–29)	26(24–29)	26(24–29)	25(22–28)	0.001
Dyslipidemia	106(42%)	117(47%)	127(50%)	101(40%)	0.103
History of myocardial infarction	13(5%)	27(11%)	26(10%)	33(13%)	0.023
Current smoker	121(48%)	94(37%)	83(34%)	54(21%)	<0.001
***Clinical data***					
Heart rate	73(64–85)	72(62–82)	80(69–90)	84(72–100)	<0.001
SBP (mmHg)	140(124–158)	139(120–157)	145(129–162)	133(119.5–157)	0.026
DBP (mmHg)	83(72–95)	80(70–90)	80(70–97)	80 (69–90)	0.01
Anterior wall infarction	80(31%)	75(29%)	83(33%)	125(49%)	0.001
LVEF (%)	60(52–66)	59(50–63)	55(46–64)	46(35–55)	<0.001
ST-elevation MI	156(61%)	140(55%)	159(62%)	167(66%)	0.085
Heart failure (Kilip class >1)	31(12%)	30(12%)	53(21%)	127(50%)	<0.001
***Acute treatments***					
β-blockers	212(83%)	212(83%)	204(80%)	172(68%)	0.001
Amiodarone	10(4%)	10(4%)	17(7%)	27(11%)	0.005
***Biological data***					
Peak CK (UI/L)	863(241–2607)	808(221–2158)	729(278–2548)	517(215–1448)	0.005
Creatinine clearance, mL/min	92(75–114)	82(63.2–105)	79.3 (57–105)	52(35–71)	<0.001

Data are presented as N(%) or median (25^th^–75^th^). P values correspond to one-way ANOVA.

LVEF: Left Ventricular Ejection Fraction.

CK: Creatine Kinase.

Nt-proBNP: N-terminal pro B type Natriuretic Peptide.

MI: Myocardial Infarction.

**Figure 1:**
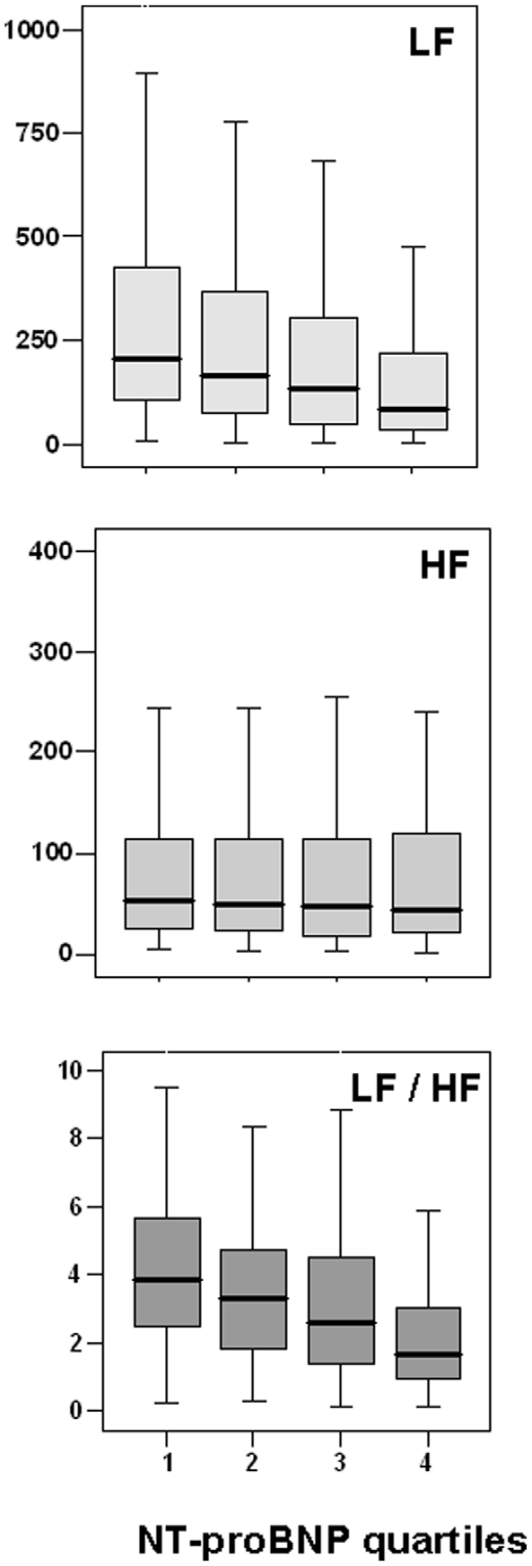
Heart rate variability parameters in spectral analysis according to of Nt-proBNP quartiles: Low Frequency (LF), High Frequency (HF) and Low frequency/High Frequency (LF/HF) ratio. P values correspond to one-way ANOVA.

HRV parameters (i.e. SDNN, LF/HF, LF, VLF and total power) gradually decreased with increasing Nt-proBNP (Q4 to Q1) (table 2). However, pNN50, rMSSD and HF values were similar whatever the propeptide quartiles (table 2). Median values for LF, HF and LF/HF according to the Nt-proBNP quartiles are presented in figure 1. Variations in the LF/HF ratio are mainly due to variations in LF, since high frequencies do not vary significantly (p = 0.172).

**Table 2:** Distribution of heart rate variability indexes according to Nt ProBNP quartiles.

	Q1	Q2	Q3	Q4	p
	N = 254	N = 255	N = 255	N = 254	
PNN50 (ms)	3.71 (0.96–10.63)	4.00 (1.01–10.76)	2.99 (0.67–9.44)	3.66 (0.88–9.76)	0.805
rMSSD (ms)	25.8 (18.0–36.7)	27.7 (18.9–38.5)	25.0 (17.7–36.3)	28.1 (18.6–41.4)	0.482
SDNN (ms)	89.7 (70.6–114.9.)	86.0 (66.0–113.9)	80.2 (59.7–100.3)	73.5 (55.1–94.9)	<0.001
TP (ms^2^)	2326 (1286.50–3830.75)	1851 (1057–3480)	1722 (733–3152.5)	1226 (569.5–2373.0)	<0.001
LF/HF	3.85 (2.52–5.70)	3.33 (1.82–4.76)	2.62 (1.41–4.5)	1.67 (0.95–3.01)	<0.001
LF (ms^2^)	412 (212–848)	330 (152–739)	267 (102–608.5)	170.5 (71.25–439.5)	0.001
HF (ms^2^)	105 (51–226)	98 (46–226)	94 (36–228)	89 (44.25–235)	0.593
VLF (ms^2^)	988 (488–1714)	787 (411–1333)	557 (290–1128)	440 (170–840)	<0.001

Data are presented as median (25^th^–75^th^). P values correspond to one-way ANOVA.

**Figure 2:**
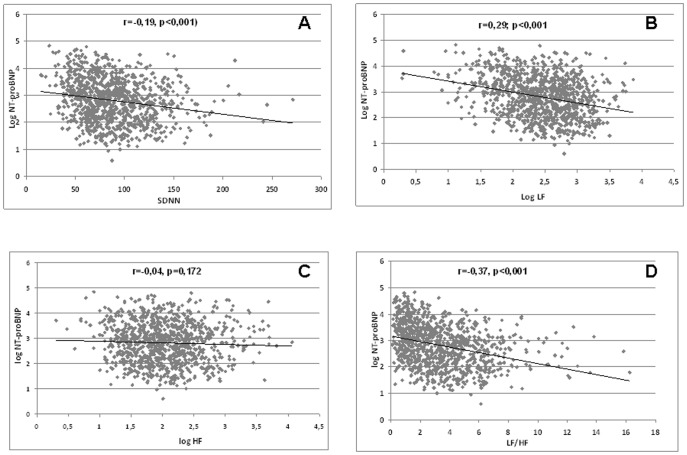
Correlation analysis between Nt-proBNP circulating levels and heart rate variability parameters on 24 h holter ECG ; A: SDNN: Standard Deviation of the NN interval (SDNN); B: Low frequency (LF); C: High frequency (HF = ; D: Low frequency/high frequency (LF/HF) ratio. P values correspond to one-way ANOVA. LF and HF are log-transformed values.

In correlation analysis, the level of Nt-proBNP was associated with SDNN (r = −0.19, p<0.001), LF/HF ratio (r = −0.37, p<0.001), and LF (r = −0.29, p<0.001) but not HF (r = −0.04, p = 0.172) (figure 2).

Multiple linear regression analysis showed that plasma levels of NT proBNP were independently associated with LF/HF, even after adjustment for confounding factors (table 3).

**Table 3:** Multivariate analysis of factors associated with Log LF/HF.

Variable	Beta	Standard Error	p
Log Nt-proBNP	−0.065	0.015	<0.001
Beta-blocker	0.062	0.026	0.020
Female	−0.127	0.025	<0.001
Age	−0.009	0.001	<0.001

## Discussion

This large prospective study in patients after MI is the first to report that high levels of Nt-proBNP at admission is strongly predictive of deterioration in HRV, as evidenced by a marked decrease in the LF/HF ratio.

### Heart rate variability determinants

HRV temporal and spectral analysis is a strong and independent prognosis marker after acute MI. [6], [7] . Although HRV early decreases after MI, HRV parameters are stable from the second to fifth day after MI. [12] In addition, HRV is a useful tool for risk stratification for both short and long term prognosis. [13] In our study, older age and women were associated with decreased LF/HF ratio, consistent with findings showing that vagal modulation diminishes with increasing age. Moreover, men have a greater HRV than women, although this difference disappears after age 50. [14]

In patients under cardiac rehabilitation program, LF/HF ratio was improved by roughly 50% in patients taking betablockers corresponding to a more favorable sympathovagal balance. [15] Propranolol treatment for 1 month induced a greater increase in HF than with placebo. [16] In addition, LF/HF ratio increased in the placebo group but decreased in the betablocker group, suggesting sympathetic inhibition. An increase in LF/HF ratio in patients under metoprolol or atenolol 3 weeks after an acute MI has also been reported. [17] In our study, most (i.e. 80%) patients were on beta-blocking therapy, resulting in a more favorable LF/HF ratio. To the best of our knowledge, no studies have clearly demonstrated the beneficial effect of beta-blockers on HRV at such early stage after the acute phase of MI.

### Sympathetic activity by spectral analysis

High frequency index (i.e. HF) reflects parasympathetic modulation, and considered as mainly influenced by respiration. LF power is probably driven by both the sympathetic and parasympathetic nervous system. Moreover, LF power is also considered as an index of sympathetic modulation of the heart rate. Although LF/HF ratio is recognized as an index of vagosympathetic balance, caution must be taken on the interpretation of this ratio, [18] because reduced LF and the LF/HF ratio may rather correspond to a decreased response of the sinus node to ANS modulations and baroreflex dysfunction, rather than an increase in sympathetic activity. In humans, LF power may also primarily reflects baroreflex modulation rather than sympathetic tone. [19] In our study, LF/HF ratio alteration across the quartiles was mostly driven by a decrease in LF, with similar HF levels. Hence, the decreased predominance of LF suggests a gradual decrease in sympathetic tone with increasing Nt-proBNP levels.

### Nt-proBNP and ANS

BNP have a wide spectrum of favorable effects including diuretic, natriuretic, and hypotensive properties, and inhibition of the Renin-Angiotensin-Aldosterone System and of endothelin secretion. Thus, infusion of BNP has been proposed as a novel therapy for heart failure. Natriuretic peptides are known to interplay with sympathetic activity, but their action remains controversial. In isolated guinea pig hearts, BNP infusion elicited norepinephrine release, via an increase in intracellular calcium and cAMP formation [20]. In contrast, in a randomized clinical trial, IV administration of ANP in patients with first anterior myocardial infarction significantly improves cardiac sympathetic nerve activity and left ventricular remodeling. [21] In animal studies, BNP infusion modified ANS activity by reinforcing reflex bradycardia. [8] Moreover, BNP infusion at doses in the physiologic range resulted in a reduction in cardiac sympathetic activity in both healthy subjects and patients with heart failure. [9] Whether this inhibitory effect is due to a direct action of the natriuretic peptide, or whether it is indirectly related to a reduction in cardiac filling pressures, remains to be determined.

The association between Nt-proBNP or BNP circulating levels and HRV remains controversial. A recent small study involving 148 healthy subjects, found that high levels of NT proBNP correlated positively with LF and the LF/HF ratio and negatively with SDNN. [22] In contrast, an increased BNP/SDNN ratio was a strong and independent predictor of hospitalization for heart failure after an acute MI [23]. In our study, the inverse relationship between the propeptide and SDNN lost significance after adjustment for confounding factors.

In patients with acute MI or heart failure, decreased HRV is a prognostic factor for mortality. [6], [7] Patients with heart failure have diminished HRV, and this decrease is also a predictor of death due to heart failure. Impaired HRV is a stronger predictor of death in patients with preserved LVEF. [24] However, the underlying pathophysiological mechanism remains unclear. Nt-proBNP is also a reliable independent predictor of sudden cardiac death after MI. [25] Heart failure is associated with neurohumoral excitation resulting in an abnormal autonomic modulation of the cardiovascular system. Hence, in patients with heart failure, LF/HF is reduced, indicating an impairment of autonomic reactivity and the attenuation of responses correlated strongly with impairement of left ventricular pomp. [26] Hence, in patients with impaired left ventricular function, the pathophysiologic mechanisms of LF/HF ratio and of reduced LF oscillations are complex [27]. Further studies are needed to specifically address the relationship between the propeptide, sympathetic nerve activity and LF oscillations in such patients.

### Study limitations

The delay between the propeptide and HRV measure HRV may influence the findings since HRV parameters are not all stable during the first two weeks after AMI. However, as shown by Carpeggiani et al, [28], the significance between admission and discharge (13±7 d) was obtained on a large paired analysis, and a large delay between the 2 HRV measures and therefore this significant increase may not apply to our study population. Moreover, LF/HF was stable during the study duration since neither LF/HF ratio nor the normalized powers did change during hospitalization (Discharge: 3±3 vs admission: 3±6). Therefore, Nt-proBNP measured at admission could predict long-term deterioration of spectral HRV balance. The main strength of this study is the use of a large regional population-based registry, reflecting the daily clinical practice in the setting of acute MI, with prospectively collected data including comprehensive analysis of all the parameters that could potentially influence HRV. This registry, however, suffers from the usual limitations of observational, non randomized studies, and therefore determines correlations, rather than causal relationships. The observation of a decreased HRV in patients with high Nt-proBNP levels must therefore be interpreted with a fair amount of caution. However, the adjustment for a wide range of possible confounding factors limits the risk of bias in our conclusions. Hence, although we cannot exclude the impact of other unmeasured confounding factors, we may think that the observed effects on HRV are robust and reliable.

## Conclusion

Our study showed for the first time a strong association between high levels of Nt- ProBNP and diminished HRV after an acute MI, even after adjustment for confounding factors. In addition, our results provide further insights on the pathophysiological effects of the propeptide, and its interplay with the sympathetic nervous system. Further experimental studies are needed to fully explore the physiological pathways involved in such effects and to determine their prognosis and therapeutic impact.
